# Analysis on the Propagation and Assembly of Metallic Nanoparticles through Subwavelength Apertures with Overlapping Electrical Double Layers

**DOI:** 10.1021/acs.jpcc.4c06715

**Published:** 2024-11-28

**Authors:** Carlos Vargas, Federico Méndez, Carlos Escobedo

**Affiliations:** † Departamento de Termofluidos, Facultad de Ingeniería, Universidad Nacional Autónoma de México, Coyoacán, Ciudad de México 04510, Mexico; ‡ Department of Chemical Engineering, Queen’s University, Kingston, Ontario K7L 3N6, Canada

## Abstract

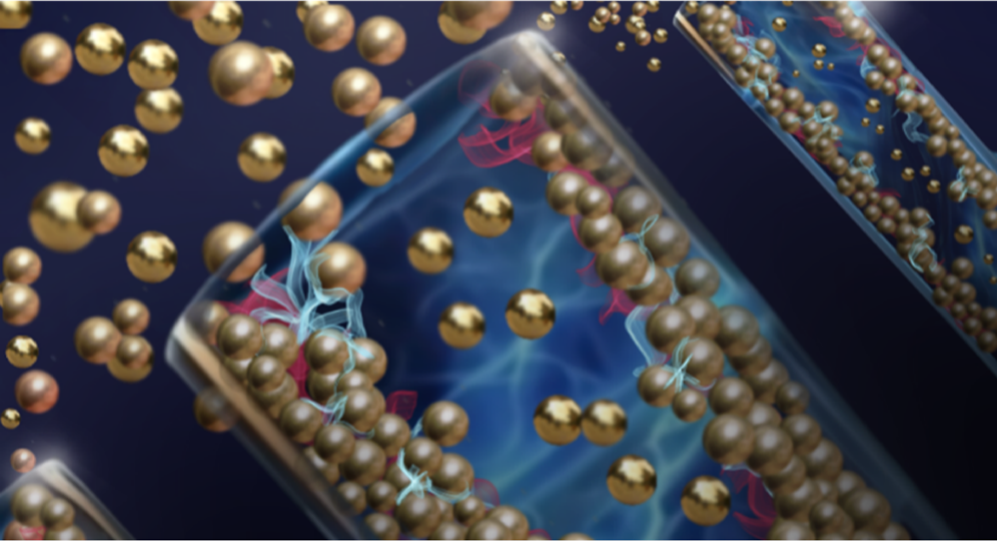

Hybrid nanoplasmonic structures composed of subwavelength apertures in metallic films and nanoparticles have recently been demonstrated as ultrasensitive plasmonic sensors. This work investigates the electrokinetically driven propagation of the assembly mechanism of the metallic nanoparticles through nanoapertures. The Debye–Hückel approximation for a symmetric electrolyte solution with overlapping electrical double layers (EDLs) is used to obtain an analytical solution to the problem. The long-term silver nanoparticle concentration response is derived using the homogenization method and a multiscale analysis. The results indicate that uncharged nanoparticles will flow through the nanohole array if the nanochannel height is larger than the Debye length (*h*_0_ > λ_D_), while a trapping mechanism occurs, due to the overlapping of the EDL, when *h*_0_ ∼ 3.8λ_D_. For charged nanoparticles, the response to the electric field occurs locally with the walls of the nanochannel, regardless of its height. For a critical value of the nanochannel length, the leading order of the concentration field becomes purely diffusive.

## Introduction

Optofluidics, the emergent field arising from the synergistic combination of photonics and microfluidics, has enabled the development of ultracompact devices with applications in different fields, such as label-free sensing.^1^ Ordered arrays subwavelength apertures, known as nanohole arrays (NHAs), have been demonstrated as nanoplasmonic sensors that can be fabricated with accurate periodicities and aperture shapes due to recent advances in nanofabrication techniques.^2^ NHAs feature relatively long channels (in the order of 10^1^ to 10^2^ nm)^3,4^ with customizable pore sizes.^5,6^ These nanoapertures have been used for fluidic transport of considerably small volumes, and the enrichment of electrocharged analytes results in improved sensor response^7−12^ and controllable nanoinjection across the nanostructured substrate. Unlike microfluidics, nanofluidics involve nanostructures where the solid boundaries are extremely close, and overlapping of electrical double layers (EDLs) is possible,^13^ which invalidates the use of Boltzmann distribution for ionic charge density.

Recent studies have presented analytical models for overlapped EDLs. Qu and Li^14^ derived a model to determine the electrical potential and ionic concentration distributions between two infinitely large flat plates, establishing corrected boundary conditions for these distributions. Golovnev and Trimper^15^ obtained an analytical solution for the Poisson–Nernst–Planck equation when Faradaic processes are discarded, revealing different ion concentration behaviors in both the short- and the long-time regimes. Zachariah et al.^16^ analyzed the repulsive forces that appear during the collapse of the EDLs using the DLVO theory, concluding that the hydration force is due to multiple layering of hydrate ions, which subsequently undergo transitions between different confined adsorbed ion states. Such a case occurs when a microchannel is connected to a nanochannel, and a micro–nano interface is formed, leading to charge transport and ion concentration polarization (ICP).^17^ ICP enables the enrichment of charged particles, such as biomarkers and ions, as well as rectification effects on ionic current.^18,19^ In this regard, Mani et al.^20^ and Yaroshchuk and Bondarenko^21^ established transport analytical models considering the area average for ICP, with emphasis on the dominance of axial diffusion in determining the extent of diffuse layers at micro-to-nanochannel interfaces when the ion reservoir is large. In the context of sensing, the transport and placement of metallic nanoparticles within and around NHAs to construct hybrid nanoplasmonic structures with improved sensitivity have been demonstrated experimentally.^11,22,23^ However, the reported analytical work has focused on ionic concentration and does not address the transport scenarios with charged colloidal systems.

In this work, we investigate the role of the EDL overlapping on both charged and uncharged metallic nanoparticles in nanoconfinement using an analytical approach. The analysis is focused on the creation of hybrid nanostructures as surface-enhanced Raman scattering (SERS) substrates, as previously reported experimentally.^11^ The investigation involves flow-through metallic nanoapertures that support surface plasmon resonance and metallic nanoparticles that naturally exhibit localized surface plasmon resonance, within a microfluidic environment under an applied electric field. We analyze the significance of the field distribution and the influence of the overlapping EDL on it, elucidating the critical impact of some parameters leading to novel perspectives on the physics of nanoparticle transport.

## Hydrodynamic Formulation

**Figure 1:**
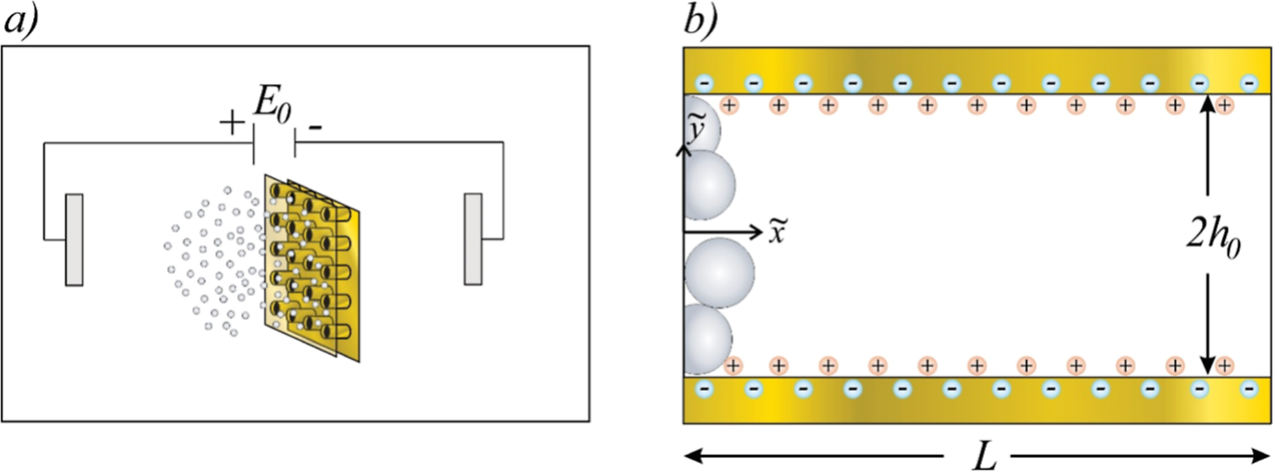
(a) Schematic representation of the microfluidic chip assembly connected to a DC source containing multiple silver nanoparticles and an NHA embedded in a gold film. (b) Close-up view of a nanohole from the NHA where silver nanoparticles respond to the induced electrical potential in the overlapped EDL.

The system used in this study encompasses a microfluidic chip assembly with an embedded metallic NHA under the influence of a direct current (DC) electric field applied externally, as shown in Figure 1a. A Newtonian fluid is assumed to contain small ions (average size ∼1 Å) and large silver nanoparticles (Ag NPs), flowing through an NHA. Both the ionic and Ag NP concentrations are initially uniform throughout the system. An electroosmotic flow (EF) emerges from an externally imposed electric field  and the induced field in the EDL. The imposed external field yields the transference of electrons to particles, resulting in the acquisition of a net negative surface electric charge. Some particles may undergo polarization while retaining their electrical neutrality; in this manner, consideration of both charged and uncharged nanoparticles in the present analysis is warranted. Figure 1b depicts a cross-sectional view of a single nanohole within the NHA. Notably, silver nanoparticles exhibit a distinct Gaussian distribution concentration close to the nanohole’s entrance, a phenomenon attributed to the localized enhancement of the electric field.^8^ The effect of the electric field gradient at the rim of the nanoholes on both the ions and the Ag NPs is of particular interest for producing hybrid nanostructures that enable SERS. This system, along with the assumption of NHA axis symmetry regarding electrokinetic phenomena, is the main domain for the analytical solution presented in this work. The nanohole is modeled as an isothermal flat nanochannel of height *h*_0_ and length *L*. A 2D Cartesian system of coordinates  is adopted at the nanochannel left inlet, where · indicates that the variable has dimensional units. The extension of the model to cylindrical coordinates is trivial, as reported by Pennathur and Santiago.^3^

### Governing Equations

The governing equations that serve as a starting point to investigate the EF in this study are the continuity equation
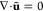
1and the Navier–Stokes equations
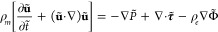
2where ρ_*m*_ is the fluid density, considered constant,  is the velocity vector,  is the time,  is the pressure, ∇ is the Nabla operator defined as , and  is the stress tensor for a Newtonian fluid, given by
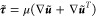
3where *μ* is the dynamic viscosity of the fluid. The hydrodynamic boundary conditions related to the impermeability and no-slip condition at the walls are given by
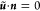
4and
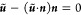
5respectively. ***n*** represents the unit vector normal to the microelectrode surface pointing toward the fluid. The pressure is  at  = 0,*L*. The electrical body force in eq 2 consists of the electric charge density ρ_*e*_ and the total electrical potential , which is governed by Poisson’s equation
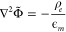
6where ϵ_*m*_ denotes the dielectric permittivity of the medium. Here,  is split into the induced nonuniform equilibrium potential in the EDL, , and the potential describing the external electric field , where *E*_*x*_ = ϕ_0_/*L* and ϕ_0_ is the voltage provided by the generator. The boundary condition for  at the walls is  = ζ, where ζ is the zeta potential. The electrical charge density ρ_*e*_ is proportional to the local concentration difference between the cations and anions. For a symmetric (*z*/*z*) electrolyte solution, the charge density can be defined as
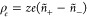
7where *z* is the valence of the electrolyte, *e* represents the fundamental charge of an electron, and  represents the concentration of cations and anions, respectively. Transport of ions in a dilute solution is described by the Nernst–Planck equation^24^
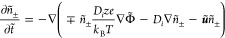
8where *D*_*i*_ is the diffusion coefficient of the ions, *k*_B_ is the Boltzmann constant, and *T* is the absolute temperature. Equation 8 is subject to the following boundary conditions^14^
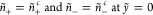
9

The concentration field  of the diffusing charged NPs is governed by the convective diffusion equation^25^
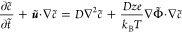
10where the molecular diffusion coefficient of nanoparticles, denoted as *D*, is determined through the Stokes–Einstein equation^26^
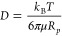
11where *R*_*p*_ is the hydrodynamic radius of the nanoparticles. In eq 10, the second right-hand term corresponds to an electromigration phenomenon, which consists of nanoparticle motion under the influence of Coulomb force. The Coulomb force appears between the gradient of the total electrical potential and the electrically charged NPs. Therefore, this term should only be considered for charged NPs. The boundary and initial conditions associated with eq 10 are
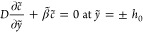
12
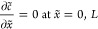
13
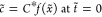
14where  is the rate of disappearance of NPs due to an irreversible first-order reaction between the solution and the walls,^27^C* is the initial concentration of NPs, , and *a* is a constant. In boundary conditions 12 we consider two cases: (i) the nonpenetration boundary for uncharged nanoparticles and (ii) the so-called perfect-sink model for charged nanoparticles. The nonpenetration boundary is obtained when  = 0, which specifies that there is no penetration of the particles at the boundary, ensuring that any change in concentration at the wall is solely due to diffusion and not advection.^28^ The boundary acting as a perfect sink, which is used in theories of diffusion of charged particles,^29^ is obtained when . This model assumes that all particles arriving at the wall will be irreversibly adsorbed immediately and subsequently disappear from the system.^30^

### Nondimensional Mathematical Model

The governing equations together with their corresponding boundary conditions can be written in a nondimensional form by introducing the dimensionless variables *x* = /L, , , , , , , ψ = /ζ, , , and . Here, *U*_*c*_ = ϵ_*m*_ζ^2^/μL is the characteristic velocity,^31^*t*_*c*_ = *L*λ_D_/*D* is the harmonic time,^32^ is the Debye length, and *n*_∞_ is the ionic number in the concentration in the bulk solution. When defining the nondimensional pressure *p*^′^, we have introduced the useful definition *P* = *p*^′^ – (1/2)(dψ/d*y*)^2^ introduced by Ajdari,^33^ which serves to eliminate the electric terms in the momentum equation in the *y* direction. Therefore, the expanded nondimensional forms of the hydrodynamic, electric, and concentration governing eqs 1–3, 6–8, and 10 are as follows
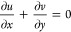
15
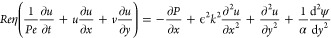
16
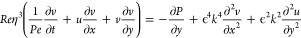
17
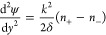
18
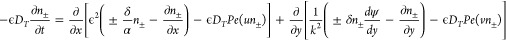
19
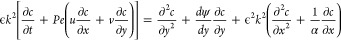
20

In eqs 15–20, ϵ = λ_D_/*L*, η = *h*_0_/*L*, *k* = *h*_0_/λ_D_, α = −ζ/ϕ_0_, δ = −ζ*ze*/*k*_B_*T*, *D*_*T*_ = *D*/*D*_*i*_, *Pe* = *U*_*c*_λ_D_/*D* is the Péclet number, and *Re* = ρ_*m*_*U*_*c*_*h*_0_/μ is the Reynolds numbers. The dimensionless boundary conditions of eqs 15–20 are
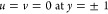
21
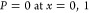
22
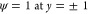
23
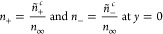
24
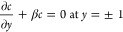
25
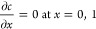
26and
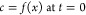
27where
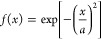
28and . In conditions 22, the induced potential gradient is zero at *x* = 0,1 due to symmetry. In nanofluidic systems, typical values of the parameters ϵ, *D*_*T*_, and *Re* are very small (λ_D_ = 10 nm, *L* = 200 nm, *D*_*i*_ = 10^–9^ m^2^/s, *D* = 8.5844 × 10^–12^ m^2^/s, *U*_*c*_ = 0.002 m/s, ϵ = 0.05, *D*_*T*_ = 10^–3^, and *Re* = 2.5 × 10^–4^), while η < 1 (η = 0.5). Therefore, a simplified version of the nondimensional governing equations, as well as a regular perturbation technique,^34^ can be used to solve the set of mentioned equations for small values of the parameter ϵ. Thus, a regular expansion is proposed for each dependent variable (say, *X*) in the following form
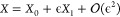
29where X = *u*, *v*, *P*, ψ. Substituting the expansion eq 29 into the nondimensional governing eqs 15–19, and collecting terms of , we obtain the following problems
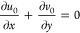
30
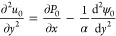
31
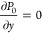
32
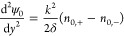
33
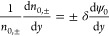
34

From eq 32, *P*_0_ = *P*_0_(*x*) should be determined as a part of the hydrodynamics problem together with *u*_0_ and *v*_0_. The solution of Nernst–Planck eq 34 is given by^14^
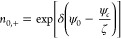
35and
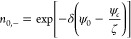
36where ψ_*c*_ is the unknown electrical potential at the center of the nanochannel. In eqs 35 and 36 it is assumed that the concentrations of cations and anions at the center are the same (*n*_0,+_ = *n*_0,–_ = 1 at *y* = 0). Further applying the Debye–Hückel approximation yields
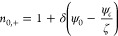
37and
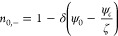
38

Substituting eqs 37 and 38 in 33 returns
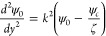
39

The  solution of Poisson eq 39, considering the boundary conditions [eq 23], is given by
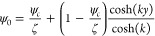
40

To recover the Boltzmann equation (ψ = 0 at *y* = 0 and *k* ≫ 1), we have assumed that ψ_*c*_ = ζ/*k*, obtaining the following simplification
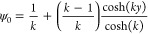
41

The general hydrodynamic solution of eq 31 is obtained by substituting eq 41 in the momentum equation and integrating the result twice with respect to *y*; considering eq 21 as the boundary condition, we obtain that the velocity profile in the lowest order is given as
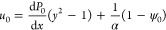
42

In the above equation, the pressure gradient is unknown and can be obtained using the continuity equation, eq 30. The suggested procedure is to substitute eqs 42 into 30, obtaining a solution for *v*_0_ and *P*_0_. However, one should find that the leading order for the variables *v*_0_ and *P*_0_ is zero. Therefore, the above physically means that there are no induced pressure terms in this order and that the velocity field is hydrodynamically developed. Therefore, substituting eqs 41 in 42 yields
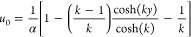
43

The next step is to assess the  solutions for ψ_1_, *u*_1_, *v*_1_, and *P*_1_. In this order, the ion concentration does not get affected by convection, thus making the  Poisson equation to yield ψ_1_ = 0, leading to *u*_1_ = *v*_1_ = *P*_1_ = 0 due to its role as the primary force in the momentum equations.

### Homogenization Method

To obtain an analytical solution for the concentration field of Ag NPs, the homogenization method^35^ is proposed to derive an expression that allows us to solve the convective diffusion equation. Thus, three distinct time scales are involved in the analysis of Ag NPs, which are as follows: the harmonic time,^32^*t*_0_ ∼ *L*λ_D_/*D*, the transverse diffusion time, *t*_1_ ∼ 4*h*_0_^2^/*D*, and the longitudinal diffusion time, *t*_2_ ∼ *L*^2^/*D*.

From typical values of the previous times (*L* = 200 nm, *h*_0_ = 100 nm, *t*_0_ = 2 × 10^–4^ s, and *t*_1_ = *t*_2_ = 4 × 10^–3^ s), the following two time scales can be introduced
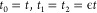
44and using eq 29 to expand for the dimensionless concentration
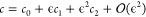
45where *c*_*j*_ = *c*_*j*_(*x*,*y*,*t*_0_,*t*_2_) and *j* = 0,1,2. The original time derivate becomes, according to the chain rule
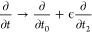
46

Substituting eqs 45 and 46 in 20 yields
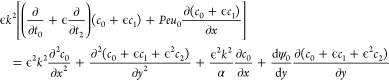
47

At order , the governing equation is given by
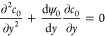
48

To obtain a solution for eq 48, we consider two cases: uncharged nanoparticles, which neglect the electromigration term (second left-hand term) in eq 48, and charged nanoparticles. In addition, both cases must satisfy the following boundary condition due to the symmetry of the nanochannel
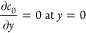
49

In both cases, the solution at  is *c*_0_ = *C*_*x*_(*x*,*t*_0_,*t*_2_). The procedure that determines the function *C*_*x*_ is given in the lines below. Taking the  from eq 47 yields the governing equation for *c*_1_
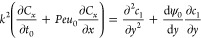
50

The next step is to take the cross-sectional average of eq 50, defined as  for any function *f*, where · will indicate the averaged function. In this context, the first right-hand term in eq 50 becomes zero as a consequence of its symmetry with respect to the *y*-axis, which is known from the inflection point at *y* = 0 [eq 49]. Similarly, the second right-hand term becomes zero due to the product of two odd functions. Thus, the cross-sectional average of eq 50 is
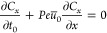
51where
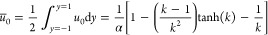
52

Substituting eqs 51 into 50 yields
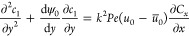
53

Considering the linearity of eq 53, the solution *c*_1_ can be expressed as
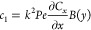
54and its substitution in eq 53 leads to a second-order ordinary differential equation for *B*(*y*) as follows
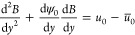
55where
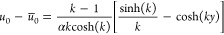
56

First, eq 55 is solved for uncharged nanoparticles using boundary condition 25 considering β = 0 as follows
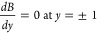
57

Solving eq 55 for uncharged NPs, neglecting the electromigration term (second left-hand term) in eq 55, yields
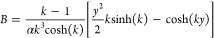
58

For charged nanoparticles (β ≫ 1), eq 55 was solved using the fourth-order Runge–Kutta method with the aid of the shooting approach together with the following boundary condition [eq 25]
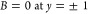
59

The  from eq 47 is given by
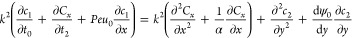
60

Substituting eqs 51 and 54 in 60 returns
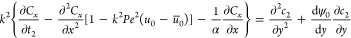
61

Taking the cross-sectional average of eq 61, the following governing equation is obtained
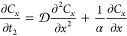
62where
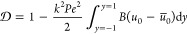
63

For uncharged nanoparticles,  was calculated by substituting eqs 56 and 58 in 63, obtaining the following equation
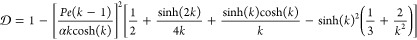
64

For charged nanoparticles,  was calculated by using numerical methods. Finally, eq 51 is added to 62, where the artifice of two times is no longer needed and can be removed^35^
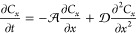
65where
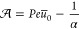
66

The second right-hand term in eq 66 is part of electromigration and should only be considered for charged nanoparticles. First, we propose a solution for *C*_*x*_ that eliminates the convective term in eq 65, i.e., the first right-hand term, as follows
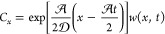
67

Substituting eqs 67 into 65 yields
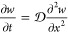
68

The initial and boundary conditions of eq 68 are taken from eqs 26–28 as follows
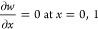
69and
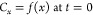
70

The general solution for the leading order, using the Fourier method for eqs 68 and substituting 67, is
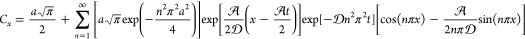
71

## Results and Discussion

In the Nondimensional Mathematical Model and Homogenization Method sections, the nondimensional potential in the EDL, velocity vector, and concentration field of silver nanoparticles, subjected to the electromigration effect, were calculated. To estimate the values of dimensionless parameters involved in the analysis, we consider values of physical and geometrical parameters that have been reported in previous work:^11^*h*_0_ = 100 nm, *L* = 200 nm, *R*_*p*_ = 25 nm, *T* = 293 K, ϵ_*m*_ = 7.8 × 10^–10^ C/V m, ρ_*m*_ = 997 kg/m^3^, μ = 1 × 10^–3^ kg/ms, ϕ_0_ = 3 V, ζ = −25.4 × 10^–3^ V, *z* = 1, *n*_∞_ = 6.022 × 10^23^ m^–3^, λ_D_ = 10 nm, *U*_*c*_ = 2.5 × 10^–3^ m/s, *D* = 8.58 × 10^–12^ m^2^/s, and *D*_*i*_ = 1.65 × 10^–9^ m^2^/s. With the previous physical domain, the dimensionless parameters for the calculations assume the following values: ϵ = 0.05, *k* = 10, η = 0.5, α = 8.4 × 10^–3^, δ = 1, *Re* = 2.5 × 10^–4^, *Pe* = 2.93, and . For the analytical process, we consider uncharged and charged Ag NPs, obtaining  and  at *k* = 10. Besides, the nondimensional concentration field is governed by the following equation
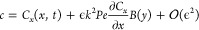
72

**Figure 2:**
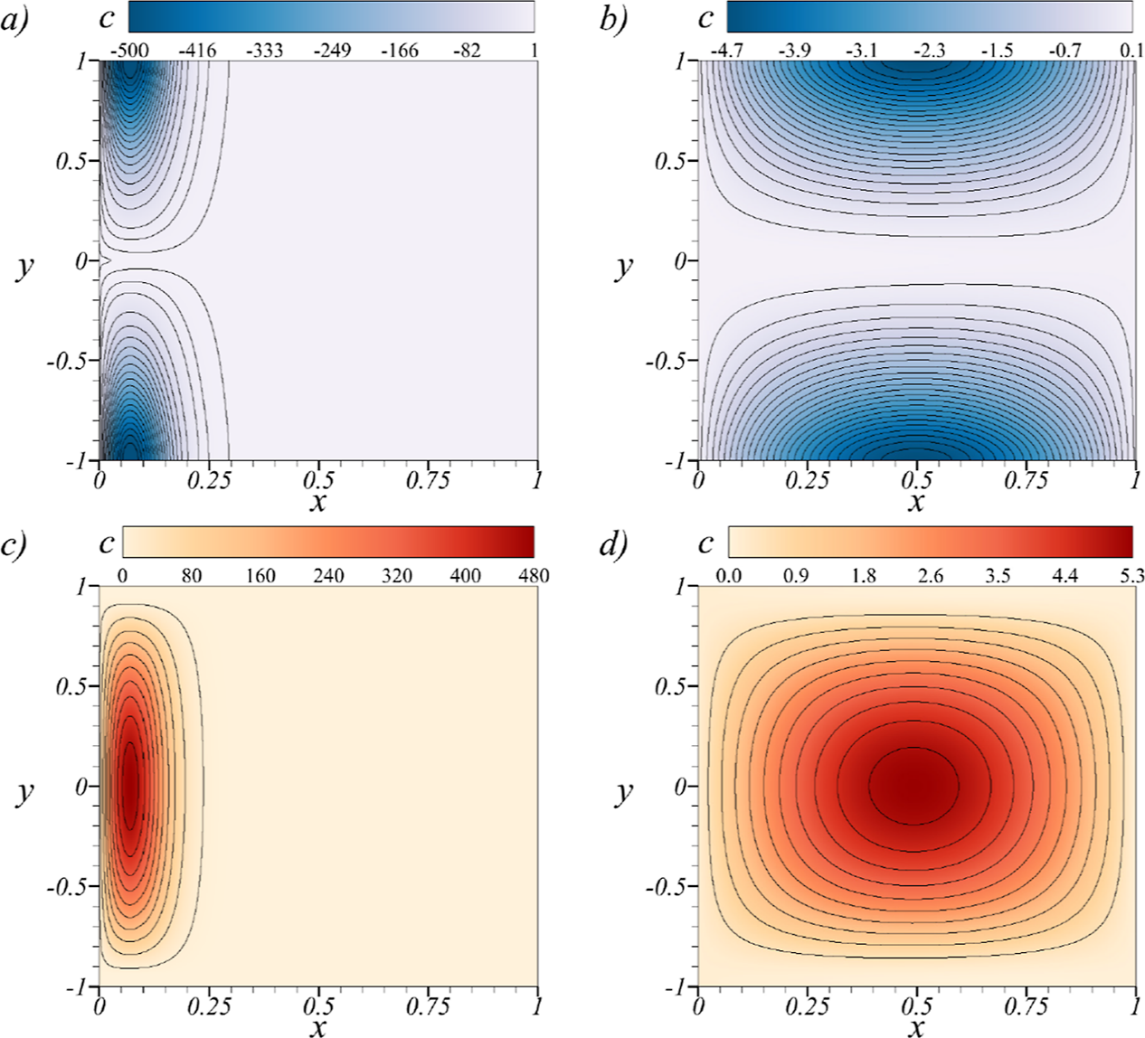
Nondimensional concentration field *c* for uncharged nanoparticles at *k* = 10, evaluated at the nondimensional time (a) *t* = 0 and (b) *t* = 10^–5^. Nondimensional concentration field *c* for charged nanoparticles at *k* = 10 and (c) *t* = 0 and (d) *t* = 10^–5^.

In Figure 2c, the nondimensional concentration field [eq 72] is shown for both charged and uncharged Ag NPs at *k* = 10. The selected times are determined using the time-dependent diffusive component in eq 71 to counteract the condition . As *t* increases beyond these selected values, the nondimensional concentration field converges to a constant value, i.e., . The first noticeable effect in Figure 2a is the propagation of NPs from their initial concentration at *t* = 0, which occurs rapidly throughout the entire system. This phenomenon is primarily attributed to diffusion, and notably, it conserves the original distribution of NPs but elongates along the system. The concentration distribution at *x* = 0.2 at this initial time is exclusively influenced by the initial boundary condition [eq 70]. Figure 2b shows the concentration field for uncharged nanoparticles at *t* = 10^–5^, where a distinctive negative concentration is observed at the walls of the nanochannel. This negative concentration indicates a deficit of nanoparticles close to the walls. On the other hand, positive concentration values at the entrance, middle, and exit of the nanochannel indicate that uncharged nanoparticles, initially located near the entrance, are driven toward the center of the channel, flow through it, and are eventually expelled at the opposite end. Figure 2d shows that the concentration field for charged nanoparticles is presented at *t* = 10^–5^. In this case, a concentration value of *c* = 0 indicates the occurrence of the reaction of NPs with the walls, as can be appreciated from eqs 13 and 59. This outcome suggests that most charged NPs react primarily at the entrance and exit regions of the nanochannel, while the excess of NPs that cannot react at the walls is concentrated at the central region. Furthermore, the coefficient , as defined in eq 66, may become zero for charged nanoparticles implying that, under certain nanochannel dimensions, no convective transport can take place for the leading order of the concentration field. An analytical expression for the critical nanochannel length, denoted as *L*_crit_, is derived by eq 66, yielding *L*_crit_ = 12π*R*_p_λ_D_^3^*n*_∞_*f*(*k*), where  [eq 52]. For instance, at *k* = 10, this results in *L*_crit_ = 474 nm. However, it is noteworthy to mention that for the current ratio , no significant changes in the concentration fields are discernible even at the critical length *L*_crit_. To improve the concentration field with convection, it is necessary to increase the  ratio. Our analysis, using eqs 64–66, reveals that this can only be achieved by increasing the parameter α and/or decreasing *k* = *h*_0_/λ_D_. The parameter α = −ζ/ϕ_0_ can be increased by subjecting the system to an external heat flux,^36^ or by reducing the applied voltage from the generator. Caution must be exercised when decreasing ϕ_0_ since this would cause a quadratic reduction in the dielectrophoretic force and thus is not recommended. Considering the reduction of *h*_0_, a lower limit of *k* = 2.5 is deduced. This requirement ensures that the height of the nanochannel allows the passage of at least one nanoparticle through it, i.e., *h*_0_ = *R*_*p*_ = 25 nm.

**Figure 3:**
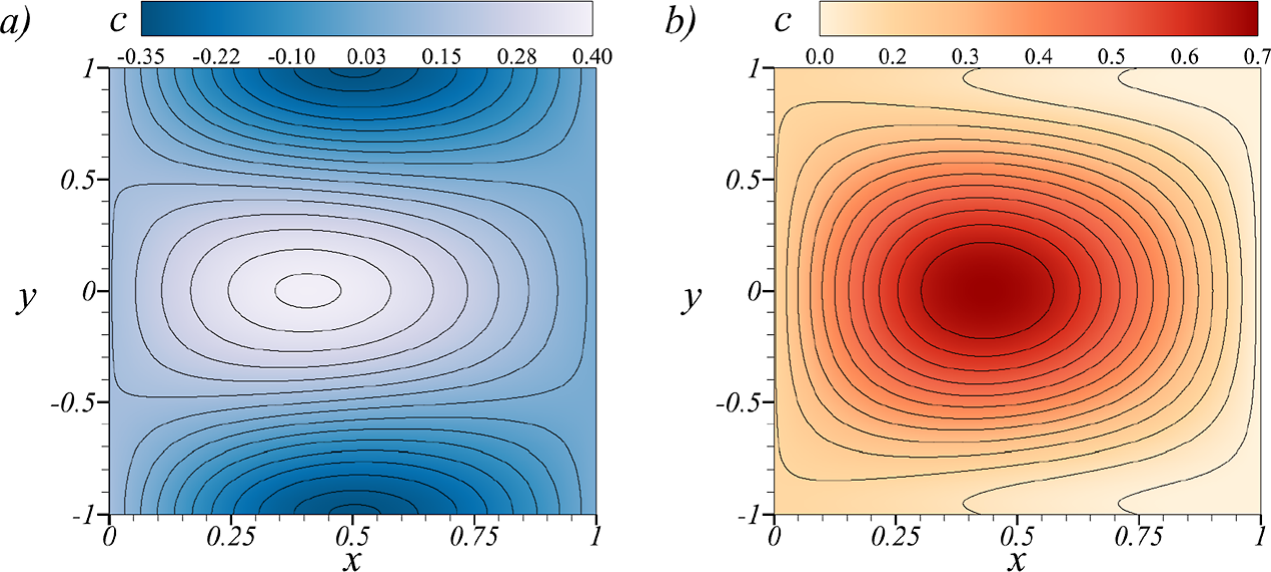
Nondimensional concentration field *c* at the nondimensional time *t* = 10^–4^ and *k* = 2.5 for (a) uncharged nanoparticles and (b) charged nanoparticles.

In Figure 3, the nondimensional concentration field at *k* = 2.5 is shown. In Figure 3a, a pronounced trapping mechanism for uncharged nanoparticles is evident, whereby a significant quantity of Ag NPs becomes trapped near the center of the nanochannel. This change in behavior is governed by the variable *B*(*y*), which, in return, is a consequence of the overlap within the EDL. This phenomenon can be elucidated by considering the representation of eqs 58 with 39 and 42, as follows
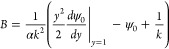
73

The trapping mechanism is observed to manifest when tanh(k) < 0.999 which is obtained when *k* = 3.8. In Figure 3b, the concentration field for charged Ag NPs is depicted, where it is observed that these nanoparticles undergo electrical reactions predominantly at the exit of the nanochannel while filling the nanohole in a counter-flow manner. For the specified value of *k* = 2.5, the critical length is calculated to be *L*_crit_ = 212 nm.

## Conclusions

Propagation of uncharged and charged nanoparticles due to an EF and electromigration in a nanochannel with overlapping EDLs has been studied by deriving an analytical expression for the ionic distribution, hydrodynamic forces, and Ag NP concentration. From the current analysis, the following major points are obtained: (i) for charged nanoparticles, colloidal transport convection is countered by electromigration, where a critical length of the nanochannel will produce a pure diffusion process for the leading concentration field solution; (ii) for uncharged nanoparticles, a trapping mechanism can be achieved due to overlapping of the EDL at *k* = 3.8; and (iii) in addition to modifying the nanochannel dimension, the propagation of colloids can be achieved by increasing the surface potential ζ through an external heat source. This last finding requires that the energy equation be coupled with the governing equations. Further studies on the propagation of colloids in nanoconfinement would be required to investigate, experimentally, the nanoaperture dimension and the variation in zeta potential. The latter could be achieved by using an external heat source^36^ or by modifying the ionic concentration of the solvent,^14^ as both approaches invalidate the Debye–Hückel approximation.

## References

[ref1] Wang J.; Maier S. A.; Tittl A. Trends in Nanophotonics-Enabled Optofluidic Biosensors. Adv. Opt. Mater. 2022, 10, 2102366 10.1002/adom.202102366.

[ref2] Escobedo C. On-chip nanohole array based sensing: a review. Lab Chip 2013, 13, 2445–2463. 10.1039/c3lc50107h. 23584239

[ref3] Pennathur S.; Santiago J. G. Electrokinetic Transport in Nanochannels. 1. Theory. Anal. Chem. 2005, 77, 6772–6781. 10.1021/ac050835y. 16255573

[ref4] Pennathur S.; Santiago J. G. Electrokinetic transport in nanochannels. 2. Experiments. Anal. Chem. 2005, 77, 6782–6789. 10.1021/ac0508346. 16255574

[ref5] Striemer C. C.; Gaborski T. R.; McGrath J. L.; Fauchet P. M. Charge- and size-based separation of macromolecules using ultrathin silicon membranes. Nature 2007, 445, 749–753. 10.1038/nature05532. 17301789

[ref6] Mukaibo H.; Wang T.; Perez-Gonzalez V.; Getpreecharsawas J.; Wurzer J.; Lapizco-Encinas B.; McGrath J. L. Ultrathin nanoporous membranes for insulator-based dielectrophoresis. Nanotechnology 2018, 29, 235704 10.1088/1361-6528/aab5f7. 29528846

[ref7] Eftekhari F.; Escobedo C.; Ferreira J.; Duan X.; Girotto E. M.; Brolo A. G.; Gordon R.; Sinton D. Nanoholes as nanochannels: Flow-through plasmonic sensing. Anal. Chem. 2009, 81, 4308–4311. 10.1021/ac900221y. 19408948

[ref8] Escobedo C.; Brolo A. G.; Gordon R.; Sinton D. Optofluidic Concentration: Plasmonic Nanostructure as Concentrator and Sensor. Nano Lett. 2012, 12, 1592–1596. 10.1021/nl204504s. 22352888

[ref9] Ertsgaard C. T.; McKoskey R. M.; Rich I. S.; Lindquist N. C. Dynamic placement of plasmonic hotspots for super-resolution surface-enhanced Raman scattering. ACS Nano 2014, 8, 10941–10946. 10.1021/nn504776b. 25268457

[ref10] Larson S.; Luong H.; Song C.; Zhao Y. Dipole Radiation-Induced Extraordinary Optical Transmission for Silver Nanorod-Covered Silver Nanohole Arrays. J. Phys. Chem. C 2019, 123, 5634–5641. 10.1021/acs.jpcc.9b00477.

[ref11] Bdour Y.; Beaton G.; Gomez-Cruz J.; Cabezuelo O.; Stamplecoskie K.; Escobedo C. Hybrid plasmonic metasurface as enhanced Raman hot-spots for pesticide detection at ultralow concentrations. Chem. Commun. 2023, 59, 8536–8539. 10.1039/D3CC01015E. 37338175

[ref12] Khosravi B.; Gordon R. Accessible Double Nanohole Raman Tweezer Analysis of Single Nanoparticles. J. Phys. Chem. C 2024, 128, 15048–15053. 10.1021/acs.jpcc.4c03536. PMC11404487 39291273

[ref13] De Leebeeck A.; Sinton D. Ionic dispersion in nanofluidics. Electrophoresis 2006, 27, 4999–5008. 10.1002/elps.200600264. 17117385

[ref14] Qu W.; Li D. A model for overlapped EDL Fields. J. Colloid Interface Sci. 2000, 224, 397–407. 10.1006/jcis.1999.6708. 10727352

[ref15] Golovnev A.; Trimper S. Analytical solution of the Poisson-Nernst-Planck equations in the linear regime at an applied dc-voltage. J. Chem. Phys. 2011, 134, 154902 10.1063/1.3580288. 21513413

[ref16] Zachariah Z.; Espinosa-Marzal R. M.; Spencer N. D.; Heuberger M. P. Stepwise collapse of highly overlapping electrical double layers. Phys. Chem. Chem. Phys. 2016, 18, 24417–24427. 10.1039/c6cp04222h. 27534602

[ref17] Yeh L.-H.; Zhang M.; Qian S.; Hsu J.-P.; Tseng S. Ion concentration polarization in Polyelectrolyte-modified nanopores. J. Phys. Chem. C 2012, 116, 8672–8677. 10.1021/jp301957j.

[ref18] Lapizco-Encinas B. H. Microscale electrokinetic assessments of proteins employing insulating structures. Curr. Opin. Chem. Eng. 2020, 29, 9–16. 10.1016/j.coche.2020.02.007.

[ref19] Wu Z.-Q.; Li Z.-Q.; Ding X.-L.; Hu Y.-L.; Xia X.-H. Influence of Asymmetric Geometry on the Ion Transport of tandem nanochannels. J. Phys. Chem. C 2021, 125, 24622–24629. 10.1021/acs.jpcc.1c06884.

[ref20] Mani A.; Zangle T. A.; Santiago J. G. On the propagation of concentration polarization from microchannel-nanochannel interfaces Part I. Analytical model and characteristic analysis. Langmuir 2009, 25, 3898–3908. 10.1021/la803317p. 19275187 PMC4816500

[ref21] Yaroshchuk A.; Bondarenko M. P. Current-Induced concentration polarization of nanoporous media: Role of electroosmosis. Small 2018, 14, 1703723 10.1002/smll.201703723. 29537135

[ref22] Chen L.; Hu C.; Dong Y.; Li Y.; Shi Q.; Liu G.; Long R.; Xiong Y. Tunable Layered Gold Nanochips for High Sensitivity and Uniformity in SERS. J. Phys. Chem. C 2023, 127, 8167–8174. 10.1021/acs.jpcc.3c01359.

[ref23] Sakamoto M.; Saitow K. Large-Area Plasmon Mapping via an Optical Technique: Silver Nanohole Array and Nano-Sawtooth Structures. J. Phys. Chem. C 2023, 127, 13105–13111. 10.1021/acs.jpcc.3c01919.

[ref24] Hille B. Ionic Channels of Excitable Membranes; Sinauer Associates Inc., 1992.

[ref25] Probstein R. F. Physicochemical Hydrodynamics; Wiley-Interscience, 2005.

[ref26] Philipse A. P. Brownian Motion. Elements of Colloid Dynamics; Springer, 2018.

[ref27] Vacík J. Electrophoresis a Survey of Techniques and Applications; Elsevier, 1979.

[ref28] Elimelech M.; Gregory J.; Jia X.; Williams R. A. Particle Deposition and Aggregation. Measurement, Modelling and Simulation; Butterworth-Heinemann, 1995.

[ref29] Leonard G.; Mitchner M.; Self S. A. Particle transport in electrostatic precipitators. Atmos. Environ. 1980, 14, 1289–1299. 10.1016/0004-6981(80)90230-9.

[ref30] Adamcyzk Z. Particle deposition from flowing suspensions. Colloids Surf. 1989, 39, 1–37. 10.1016/0166-6622(89)80176-3.

[ref31] Miloh T.; Boymelgreen A. Travelling wave dipolophoresis of ideally polarizable nanoparticles with overlapping electric double layers in cylindrical pores. Phys. Fluids 2014, 26, 072101 10.1063/1.4884956.

[ref32] Bazant M. Z.; Thornton K.; Ajdari A. Diffuse-charge dynamics in electrochemical systems. Phys. Rev. E:Stat., Nonlinear, Soft Matter Phys. 2004, 70, 021506 10.1103/PhysRevE.70.021506. 15447495

[ref33] Ajdari A. Electro-osmosis on Inhomogeneously charged surfaces. Phys. Rev. Lett. 1995, 75, 755–758. 10.1103/PhysRevLett.75.755. 10060106

[ref34] Bender C. M.; Orzag S. A. Advanced Mathematical Methods for Scientists and Engineers I: Asymptotic Methods and Perturbation Theory; Springer Science and Business, 2013.

[ref35] Mei C. C.; Vernescu B. Homogenization Methods for Multiscale Mechanics; World Scientific Publishing Co., 2010.

[ref36] Vargas C.; Bautista O.; Méndez F. Effect of temperature-dependent properties on electroosmotic mobility at arbitrary zeta potentials. Appl. Math. Model. 2019, 68, 616–628. 10.1016/j.apm.2018.11.050.

